# Unfolded protein response to autophagy as a promising druggable target for anticancer therapy

**DOI:** 10.1111/j.1749-6632.2012.06739.x

**Published:** 2012-10-10

**Authors:** Dong Hoon Suh, Mi-Kyung Kim, Hee Seung Kim, Hyun Hoon Chung, Yong Sang Song

**Affiliations:** 1 Department of Obstetrics and Gynecology, Seoul National University College of MedicineSeoul, Republic of Korea; 2 Cancer Research Institute, College of Medicine, Seoul National UniversitySeoul, Republic of Korea; 3 Department of Agricultural Biotechnology, Seoul National UniversitySeoul, Republic of Korea

**Keywords:** endoplasmic reticulum stress, unfolded protein response, autophagy, cancer, molecular targeted therapies

## Abstract

The endoplasmic reticulum (ER) is responsible for protein processing. In rapidly proliferating tumor cells, the ER tends to be overloaded with unfolded and misfolded proteins due to high metabolic demand. With the limited protein-folding capacity of the ER, tumor cells often suffer from more ER stress than do normal cells. Thus, cellular stress responses to cope with ER stress, such as the unfolded protein response (UPR) and autophagy, might be more activated in cancer cells than in normal cells. The complex signaling pathways from the UPR to autophagy provide promising druggable targets; a number of UPR/autophagy-targeted anticancer agents are currently in development in preclinical and clinical studies. In this short review we will discuss the potential anticancer efficacy of modulators of cellular stress responses, especially UPR and autophagy, on the basis of their signaling pathways. In addition, the current developmental status of the UPR/autophagy-targeted agents will be discussed.

## Introduction

The endoplasmic reticulum (ER) is an organelle that is responsible for protein folding and assembly, lipid and sterol biosynthesis, and free calcium storage.^1^ Many types of cancers have been demonstrated to rely on the ER to correctly maintain the structure of the important proteins of key signaling pathways.^2^ The ER of rapidly proliferating tumor cells is flooded with an enormous amount of protein because the increased metabolic activities of cancer cells are executed through the activation of diverse signaling pathways.^3^–^5^ Thus, the high proliferation rate of cancer cells requires increased activity by the ER machinery in facilitating protein folding, assembly, and transport. However, the capacity of the ER to process proteins is limited and the accumulation of unfolded and misfolded proteins could lead to ER stress in cancer cells.^6^ ER stress can be caused by pathologic stimuli, such as nutrient deprivation, oxidative stress by reactive oxygen species (ROS), or energy perturbation, conditions that are commonly encountered by most solid tumors.^6^,^7^ Upon ER stress, a variety of human cancers activate a group of signal transduction pathways, inducing cellular stress responses such as the unfolded protein response (UPR) and autophagy to maintain ER homeostasis.^8^,^9^

The UPR is a series of complementary adaptive mechanisms to cope with protein-folding alterations.^10^ Initially, the UPR is intended to reestablish homeostasis and normal ER function in the cell by blocking protein translation and activating the signaling pathways that lead to increased production of molecular chaperones involved in protein folding.^11^,^12^ However, when the adaptive mechanisms fail to restore normal ER function due to protracted or excessive stress stimuli, the UPR pathways may initiate apoptotic pathways to remove the stressed cells.^13^

In addition, it is becoming increasingly clear that ER stress can also lead to the induction of autophagy.^9^ The autophagy is a self-eating homeostatic, catabolic process, regulated by the autophagy-related gene (ATG). Tumor cells may activate autophagy in response to cellular stress and increased metabolic demands related to rapid cell proliferation. Cellular proteins and organelles are engulfed by autophagosomes, digested in lysosomes, and recycled to sustain cellular metabolism.^14^ Therefore, autophagy can promote cell survival during times of nutrient deprivation and hypoxia. However, it has been reported that autophagy is associated with the induction of nonapoptotic cell death when protein and organelle turnover overwhelm the capacity of the cell, despite the sustained activation of autophagy.^14^

The respective roles of the UPR and autophagy as important cellular stress responses in different forms of cancer seem to be complex and even conflicting depending on the duration and intensity of the stress stimuli. Although the functions of the UPR and autophagy in tumorigenesis have not yet been fully characterized, many studies have focused on the UPR and autophagy as novel therapeutic targets for anticancer therapy because of the different metabolic status and dependence on stress responses between normal and cancer cells.^6^

In this review we will discuss the potential anticancer efficacy of modulators of cellular stress responses, especially UPR and autophagy, on the basis of their signaling pathways. In addition, the current developmental status of the UPR- and autophagy-targeted agents will be thoroughly explored.

## Signaling pathways from the UPR to autophagy

### UPR and ER stress-induced apoptosis

**Figure 1:**
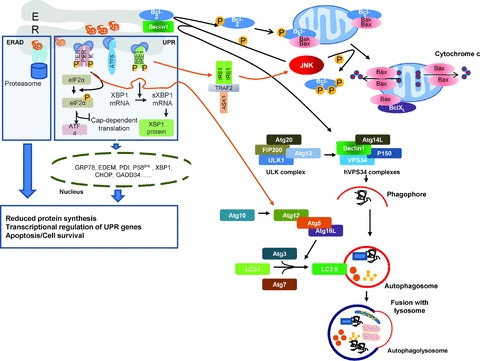
Cell-signaling pathways from the UPR to autophagy and ER stress-induced apoptosis. Conditions of ER stress where unfolded or misfolded proteins build up cause GRP78 to release three major ER stress sensors on the ER membrane: PERK, ATF6, and IRE1, which are then activated. Upon release from GRP78, IRE1 oligomerizes, autophosphorylates, and processes XBP1 mRNA to produce an active transcription factor, spliced XBP1 (sXBP1). sXBP1 activates stress-inducible genes involved in protein folding and protein degradation, including the genes ER degradation-enhancing alpha-mannosidase-like protein (EDEM), protein disulphide isomerase (PDI), and X box-binding protein 1 (XBP1). Active ATF6 translocates to the nucleus and induces the expression of genes with ER response elements in their promoters, including CHOP and XBP1. Activated PERK dimerizes and autophosphorylates itself. Activated PERK phosphorylates and inactivates eIF2α, which suppresses global cap-dependent mRNA translation, but activates ATF4 translation. ATF4 translocates to the nucleus and induces the transcription of genes for amino acid metabolism, redox reactions, CHOP, and GADD34. These responses reduce the unfolded protein load in the ER by reducing the global protein synthesis, by increasing the folding capacity of the ER and by removing misfolded proteins from the ER. Largely through the two pathways of the UPR, the PERK-eIF2α and IRE1-TRAF2-JNK pathways, ER stressors can induce autophagy (orange arrow). Activation of the PERK-eIF2α axis of the UPR pathways was shown to upregulate Atg12, convert LC3-I to LC3-II, and subsequently facilitate autophagosome formation.^27^ Activated IRE1α can recruit tumor-necrosis factor receptor associated factor 2 (TRAF2) and apoptosis-signal regulating kinase (ASK1), subsequently activating JNK. Severe ER stress leads to activation of JNK that downregulates the anti-apoptotic protein Bcl-2 by phosphorylating Bcl-2 on the mitochondrial and ER membrane. JNK-mediated phosphorylation of Bcl-2 releases Beclin1 from its inhibitory interaction with Bcl-2 at ER membrane. Freed Beclin1 induces autophagy through the formation of hVPS34 complexes. The first step of autophagy (induction) is activated by ULK complex composed of ULK1, Atg13, FIP200, and Atg20. The nucleation step is mediated by a complex involving VPS34 (also known as PI3KCIII) with either Beclin1-Atg14L-VPS34-p150 or Beclin1-UVRAG-VPS34-p150. The elongation of the phagophore is mediated by two ubiquitin-like conjugation systems that together promote the assembly of the Atg5-Atg12-Atg16L complex and the processing of LC3. The lipidated form of LC3-I (LC3-II) is attached to both faces of the phagophore membrane. ER stress can induce apoptosis through an intrinsic pathway involving cytochrome c release from mitochondria and caspase activation. Autophagy is also induced via JNK activation that releases Beclin1 from its inhibitory interaction with Bcl-2 at the level of ER, via Bcl-2 phosphorylation. UVRAG, UV radiation resistance associated gene protein; VPS, vacuolar protein sorting; ERAD, ER-associated degradation. Modified and adapted by permission from Nature Publishing Group from Ref. 29.

There are three major ER stress sensors, such as pancreatic ER kinase (PKR)-like ER kinase (PERK), activating transcription factor-6 (ATF6), and inositol-requiring enzyme 1 (IRE1) (Fig. 1). Upon accumulation of unfolded or misfolded proteins in the ER, PERK, ATF6, and IRE1 may be sequentially activated following their dissociation from the ER chaperone GRP78. Activated PERK transiently inhibits protein synthesis by phosphorylating eukaryotic initiation factor 2α (eIF2α), which suppresses general cap-dependent mRNA translation, with the exception of *ATF4* mRNA. ATF4 translocates to the nucleus and induces the transcription of genes for amino acid metabolism, redox reactions, C/EBP homologous protein (CHOP), and growth arrest, and DNA damage–inducible protein 34 (GADD34). Activation of PERK also leads to the induction of CHOP, which switches the ER stress response from proadaptive to proapoptotic signaling.^15^ ATF6 is activated by proteolysis mediated by proteases S1P and S2P after its translocation from the ER to the Golgi apparatus.^16^ After translocation to the nucleus, activated ATF6 regulates the expression of ER chaperones (e.g., GRP78 and GRP94) as well as X box-binding protein 1 (XBP1) and protein disulphide isomerase (PDI) to facilitate protein folding, secretion, and degradation in the ER.^17^ IRE1α processes *XBP1* mRNA to produce an active transcription factor, spliced XBP1 (sXBP1). sXBP1 activates the transcription of the genes encoding proteins involved in protein folding, ER-associated protein degradation (ERAD), and protein quality control.^10^

Persistent or severe ER stress can induce apoptotic cell death.^18^ CHOP and c-Jun N-terminal kinase (JNK) are reported to play important roles in the induction of cell death.^19^ After transcriptional activation by ATF4, CHOP downregulates the antiapoptotic protein Bcl-2, and upregulates some BH3-only proteins and GADD34, a protein phosphatase 1 (PP1)-interacting protein that causes PP1 to dephosphorylate eIF2α and thus releases the translational suppression.^20^ JNK phosphorylates Bcl-2 and BH3-only proteins to promote apoptosis. It has been also suggested that activated IRE1α can recruit tumor-necrosis factor receptor associated factor 2 (TRAF2), which activates procaspase-4 as a mitochondria-independent apoptotic response.^21^ The IRE1-TRAF2 complex formed during ER stress can recruit the apoptosis-signal regulating kinase (ASK1).^19^ Nishitoh *et al*. demonstrated that overexpression of ASK1-induced cell death in several cell types, highlighting the importance of ASK1 in ER stress-induced apoptosis.^22^ Activation of JNK is known as a common response to many forms of stress, including ER stress, where the activation of JNK was shown to be IRE1 and TRAF2 dependent.^23^,^24^ IRE1, once activated, initially aids the UPR. However, if ER stress persists, IRE1 facilitates apoptosis by recruiting ASK1 and JNK.^19^

### ER stress–induced autophagy

Autophagy is a multistep process of sequestration and subsequent degradation of large protein aggregates and damaged organelles in autophagosomes: induction, nucleation, elongation, and completion.^25^ The induction is activated by Unc-51–like kinase 1/2 (ULK1/2) complexes, which are inhibited by mammalian target of rapamycin (mTOR). The nucleation is mediated by Beclin1-VPS34 (PI3KIII)-P150 core complexes. Ubiquitin-like protein conjugation is required at the elongation phase, which is mediated by Atg3, Atg5, Atg7, microtubule-associated protein 1 light chain 3 (LC3), Atg10, Atg12, and Atg16L to fully encapsulate the cytosolic cargo.^25^ After the completion of autophagosome formation, most of the Atg proteins (except LC3-II) on the luminal membrane are recycled in the cytosol.^14^ LC3-II remains on mature autophagosomes until fusion with lysosomes is completed and forms autophagolysosomes (Fig. 1).

### Interplay of apoptosis and autophagy

It has been demonstrated that apoptotic pathways are frequently disabled in human cancers.^26^ Both apoptotic and autophagic pathways have been reported to share mediators, which supports that there might be crosstalks between them.^26^ Therefore, autophagy could be an alternative mode of cell death in apoptosis-defective cancer cells.^14^ Activation of the PERK-eIF2α axis of the UPR pathways was shown to upregulate Atg12, convert LC3-I to LC3-II, and subsequently lead to autophagosome formation.^27^ The IRE1-TRAF2-JNK pathway of the UPR was also reported to be important for the induction of ER stress-induced autophagy (Fig. 1). It has been demonstrated in IRE1α-deficient mouse embryonic fibroblasts (MEFs) that accumulation of LC3-positive vesicles triggered by thapsigargin, an inhibitor of ER Ca^2+^-ATPase, is dependent on IRE1.^28^ Thapsigargin-induced accumulation of LC3-positive vesicles was also completely inhibited in MEFs deficient for TRAF2, a known adaptor molecule between IRE1 and JNK. Finally, the effective inhibition of LC3 translocation by JNK inhibitor suggests that the IRE1-TRAF2-JNK pathway might be essential for the induction of autophagy in MEFs challenged with ER stressors.^29^ Autophagy can lead to cell death through the interaction between Beclin1 and regulators of apoptosis such as Bcl-2/Bcl-X_L_ and BH3-only proteins.^30^ ER stress can induce apoptosis through an intrinsic pathway involving cytochrome c release from mitochondria and caspase activation. A positive feedback loop involves caspase-dependent cleavage of Beclin1. Cleaved Beclin1 then relocates to the mitochondria to enhance cytochrome c release by releasing proapoptotic proteins like Bax/Bak from the inhibitory interactions with antiapoptotic proteins like Bcl-X_L_. BH3-only proteins also liberate Beclin1 from Bcl-2 localized on the ER. Freed Beclin1 then induces autophagy.^31^ Small-molecule inhibitors of Bcl-2/Bcl-X_L_, also known as BH3 mimetics (ABT-737/263, obatoclax), can competitively disrupt the Beclin1-Bcl-2/Bcl-X_L_ interaction to trigger autophagy.^32^

## Supporting evidence for therapeutic potential of targeting the UPR and autophagy in cancer

Although several recent studies have indicated the tumor-suppressive roles of the UPR and autophagy, there is considerable evidence that supports prosurvival effects of the UPR and autophagy in cancer.

### The UPR and cancer

Recent studies have shown that at least one branch of the UPR is activated in a variety of human cancers. For example, GRP78 has been reported to be implicated in various aspects of cancer progression, including increased proliferation, evasion of apoptosis, angiogenesis, metastasis, and chemoresistance, although it is not clear whether the overexpression of GRP78 in many tumors actually contributes to these malignant phenotypes.^6^,^33^,^34^ GRP78 has also been shown to protect dormant tumor cells from chemotherapy-induced apoptosis, mainly through suppressing activation of the apoptotic pathway.^35^ Increased expression of GRP78 was observed in hepatocellular carcinoma (HCC) tissues with higher expression in moderately to poorly differentiated tissues than in well-differentiated ones.^36^

The IRE1/XBP1 axis is another mediator of the UPR that has been demonstrated in a number of studies to be important for tumor growth under stress conditions in various human cancers including breast and HCC.^37^ In an experimental study with a severe combined immunodeficiency (SCID) mouse xenograft model, XBP1-deficient transformed mouse fibroblasts were reported to have significantly reduced ability to grow.^38^ By contrast, sustained sXBP1 overexpression in Eμ-sXBP1 transgenic mice has been shown to produce features of human multiple myeloma.^39^ Scriven *et al*. demonstrated that *in vitro* UPR activation by glucose deprivation in breast cancer cells could induce chemoresistance.^40^ They also showed that estrogen stimulation might induce the overexpression of GRP78 and XBP1, both of which were used as UPR activation markers in their study. PERK/eIF2α/ATF4 has also been implicated in increased proliferation and survival of hypoxic cancer cells.^41^,^42^ Tumors derived from PERK^−/−^ mouse embryonic fibroblasts have limited ability to stimulate angiogenesis.^43^ Supporting this notion, Bi *et al*. demonstrated a functional link between the activation of the PERK/eIF2α/ATF4 pathway and the upregulation of vascular endothelial growth factor-A (VEGF-A) transcription.^41^

### Autophagy and cancer

Accumulating evidence has demonstrated that the activation of autophagy after ER stress might be cytoprotective or cytotoxic depending on the duration and degree of the stress.^7^ Autophagic degradation of preexisting intracellular proteins is a major source of free amino acid pools for survival in stressed cells.^44^ Thus, autophagy in cancer cells can confer stress tolerance, which serves to maintain tumor cell survival.^14^ In cancer cells that survive chemotherapy or radiation, activation of autophagy maintained a dormant state in residual cancer cells, which may cause tumor recurrence and progression,^45^ whereas inhibition of autophagy in many types of tumor cells has been shown to enhance the efficacy of anticancer drugs.^14^ A recent study showed that human cancer cell lines bearing activating mutations in *Ras* oncogene commonly had high basal levels of autophagy even in the presence of abundant nutrients.^46^ They also suggested that blocking autophagy in tumors could be an effective treatment approach in autophagy-addicted Ras-driven cancers. In accordance with these findings, malignant melanomas showed increased expression of LC3 compared with the early melanoma *in situ* lesions and normal melanocytes, suggesting the survival advantage provided by autophagy in the late stage of tumorigenesis.^47^ Elevated Beclin1 expression predicted the poor prognosis in patients with nasopharyngeal cancer who were treated with chemoradiation.^48^ However, studies on the expression of autophagy-related proteins in cancer tissues have shown inconsistent results. Loss of the *Beclin1* gene was shown to be associated with worse prognosis in patients with various solid tumors, including colon,^49^ squamous cell carcinoma of esophagus,^50^ and breast cancer.^51^

## Modulators of the UPR and autophagy as anticancer agents: current developmental status

Proliferating cancer cells might constitutively suffer from high oxidative/metabolic stress, whereas normal cells might not. Increased cellular stress and consequently activated cellular stress response pathways such as the UPR and autophagy pathways in cancer cells have been proposed as the underlying mechanisms by which UPR/autophagy-targeted agents could be developed as an effective anticancer strategy with the selectivity for cancer cells.

There have been two approaches suggested to modulate the cellular stress responses: inhibition of the basal activity of stress responses to prevent cells from adapting to stressful conditions and, the other approach, induction of stress stimuli to overload the machinery of stress responses tipping the balance toward cell death.^6^,^7^ There are several anticancer agents under development that affect stress response pathways although it may not be their primary mechanisms of action (Table 1). Many of them entered preclinical and phase I/II clinical trials and have been shown to induce components of stress response pathways in most cases (Table 2). Inhibitors of stress responses are also promising in cancer therapy and are under development mainly in preclinical studies. While developing those agents, it is also important to determine whether they actually induce or inhibit the stress response pathways and how the modulation of those pathways affects cell fate. Induction or inhibition of some components does not necessarily mean increased or decreased stress response pathways. In addition, since there is crosstalk between the UPR and autophagy, the consequences of the developed agents may not be predictable. For example, modulating ER stress responses may result in cell survival through autophagy instead of inducing apoptotic cell death. However, the major drawback to assessing stress response pathways is that there are no valid biomarkers to measure those dynamic processes.

**Table 1:** Drugs modulating cellular stress responses

Class	Effect on UPR or target	Drug
**ER stress inducers**		
Proteasome inhibitor	Phosphorylation of eIF2α	Bortezomib^52^
	Induction of XBP1 splicing	
	Activation of ATF4	
	PERK phosphorylation	
	CHOP induction	
HSP90 inhibitors	GRP78 induction	17-AAG^59^
	Induction of XBP1 splicing	17-DMAG
	CHOP induction	
	Activation of ATF6	
HIV protease inhibitors	CHOP induction	Ritonavir^61^
	GRP78 induction	Nelfinavir^60^
ADP ribosylation factor inhibitor	GRP78 induction	Brefeldin A^62^
	Induce all three branches of the UPR	
	ER dilation	
Leakage of Ca^2+^ from ER into cytosol	GRP78 induction	2,5-dimethyl-celecoxib^6^
	CHOP induction	
	Inhibition of protein synthesis	
Inhibitor of sarcoplasmic Ca^2+^ ATPase	Induce all three branches of the UPR	Thapsigargin^6^
**Inhibitors of ER stress response**		
GRP78 inhibitor	Inhibits induction of GRP78	Versipelostatin^63^
	Repress production of ATF4	
	Repress production of spliced XBP1	
**Autophagy inducers**		
mTOR inhibitors	mTOR	Sirolimus^68^
		Temsirolimus^64^
		Everolimus^65, 66^
		NV-128^84^
Proteasome inhibitors	Proteasome	Bortezomib^55^
		NPI-0052^56^
		Epoxomicin^57^
Tyrosine kinase inhibitors	KIT, BCR-ABL, PDGFR	Imatinib^82^
	BCR-ABL, SRC	Dasatinib^83^
	VEGFR, RAF, KIT, PDGFR, FLT3	Sorafenib^89^
HDAC inhibitors	HDAC	Vorinostat^69^
		Panobinostat^70^
Monoclonal antibodies	CD20	Rituximab^92^
	EGFR	Panitumumab^93^
Hormone treatment	Hormone receptors	Tamoxifen^85^
		Toremifene
Farnesyltransferase inhibitors	Farnesyltransferase	Lonafarnib^86^
PARP inhibitors	PARP1	ABT-888^87^
Others	Analog of vitamin D	EB1089^88^
	Antioxidant	Resveratrol^80^
	BCL2 inhibitor	GX15-070^89^
	Glycolysis inhibitor	2-deoxyglucose^90^
**Autophagy inhibitors**		
Aminoquinolines	Inhibition of lysosomal degradation	Chloroquine^94^
Others	Inhibition of lysosomal degradation	Hydroxychloroquine
	Inhibition of autophagosome formation	Quinacrine^91^
		3-methyladenine^85^

UPR, unfolded protein response; HSP, heat shock protein; XBP1, X box-binding protein 1; CHOP, C/EBP homologous protein; ATF4, activating transcription factor-4; 17-AAG, 17-Allylamino-17-demethoxygeldanamycin; 17-DMAG, 17 (Dimethylaminoethylamino)-17-demethyoxygeldamycin; PERK, pancreatic ER kinase (PKR)-like ER kinase; mTOR, mammalian target of rapamycin; HDAC, histone deacetylase; EGFR, epidermal growth factor receptor; PARP, poly(ADP-ribose) polymerase. Modified and adapted with permission from Refs. 6 and 25.

**Table 2:** Clinical studies of ER stress inducers and autophagy inhibitors in anticancer therapy

Drugs	Trial no.	Cancer types	Phase	Status
**ER stress inducers**				
HSP90 inhibitor (17-AAG)	NCT00088374	Kidney tumors	II	Completed
	NCT00096109	Breast cancer	II	Completed
	NCT00118092	Prostate cancer	II	Completed
	NCT00093821	Leukemia, sarcoma	I	Completed
	NCT00117988	Lymphoma	II	Completed
HSP90 inhibitor (IPI-504)	NCT00564928	Prostate cancer	II	Completed
	NCT00817362	Breast cancer	II	Completed
	NCT01362400	NSCLC	II	Recruiting
	NCT01427946	NSCLC	Ib/II	Recruiting
HSP90 inhibitor + proteasome inhibitor	NCT00096005	Lymphoma, solid tumor	I	Completed
	NCT00923247	Solid tumors	I/II	Recruiting
Proteasome inhibitor (Bortezomib)	NCT00428545	Solid tumors	I	Recruiting
	NCT01132911	Solid tumors	I	Completed
HIV protease inhibitors	NCT01164709	Hematologic cancer	I	Recruiting
	NCT00436735	Solid tumors	I	Active, not recruiting
	NCT01065844	Head and neck cancer	II	Recruiting
Thapsigargin	NCT01056029	Solid tumors	I	Recruiting
**Autophagy inhibitors**				
Hydrochloroquine	NCT01292408	Breast cancer	II	Recruiting
	NCT01506973	Pancreatic cancer	I/II	Recruiting
	NCT01206530	Colorectal cancer	I/II	Recruiting
	NCT00969306	SCLC	I/II	Recruiting
	NCT00933803	NSCLC	I/II	Active, not recruiting
	NCT00765765	Breast cancer	I/II	Terminated
	NCT01144169	Renal cell carcinoma	I	Recruiting
	NCT01006369	Colorectal cancer	II	Recruiting
Hydrochloroquine + mTOR inhibitor	NCT00909831	Solid tumors	I	Recruiting
Hydrochloroquine + HDAC inhibitor	NCT01023737	Solid tumors	I	Recruiting

HSP, heat shock protein; 17-AAG, 17-Allylamino-17-demethoxygeldanamycin; NSCLC, non-small cell lung cancer; SCLC, small cell lung cancer; mTOR, mammalian target of rapamycin; HDAC, histone deacetylase. Data from http://www.clinicaltrial.gov.

Here, we will briefly introduce several agents currently in development affecting stress response pathways.

### Proteasome inhibitors

Proteasome inhibitors have been shown to induce ER stress by interfering with ERAD and causing accumulation of misfolded proteins in the ER lumen. Bortezomib, which was approved for the treatment of relapsed multiple myeloma, has been demonstrated to induce several UPR proteins, including PERK, ATF4, and CHOP, resulting in increased myeloma cell death.^52^ Bortezomib was also reported to sensitize pancreatic cancer cells to ER stress-mediated apoptosis and to enhance the anticancer activity of cisplatin via JNK-dependent mechanism.^53^ However, in the latter study, bortezomib inhibited PERK and subsequent phosphorylation of eIF2α, while it induced expression of CHOP and GRP78/BiP.^54^ On the other hand, proteasome inhibitors are also known as an autophagy inducer. Bortezomib was shown to induce autophagy in colorectal cancer cells.^55^ The finding that proteasome-induced apoptosis is inhibited by inhibiting autophagy in neoplastic but not in normal cells suggests that maximum antitumor effect could be achieved by the combination of proteasome inhibitor and autophagy inhibitor. Similar findings were observed in the study by Zhu *et al*.,^56^ who demonstrated that the inhibition of proteasome in prostate cancer cells by NPI-0052 could facilitate autophagy through an eIF2α-dependent mechanism that upregulated transcription of ATG genes. They also showed that the combination of autophagy and proteasome inhibition could result in more cell death than the inhibition of either pathway alone. Similarly, autophagy-defective cells (*Belclin1*^+/−^) exhibited increased sensitivity to the proteasome inhibitor epoxomicin compared with wild-type cells (*Belclin1*^+/+^).^57^ These findings suggest that the induction of autophagy might be necessary to compensate for impaired proteasome function.

### HSP90 inhibitors

Heat shock protein 90 (HSP90) is a chaperone that is responsible for proper folding and stabilization of a large number of proteins involved in many different cellular processes. HSP90 was reported to modulate UPR through stabilizing the cytoplasmic domains of IRE1 and PERK.^58^ HSP90 inhibitors include 17-allylamino-17-demethoxygeldanamycin (17-AAG) and 17 (dimethylaminoethylamino)-17-demethyoxygeldamycin (17-DMAG), which have entered phase I/II clinical trials in lymphoma, breast, and prostate cancers. 17-AAG treatment to myeloma cells induced splicing of XBP1, upregulation of CHOP, and activation of ATF6, and induced cell death with activation of JNK and caspase cleavage, indicating that HSP90 inhibitors induce myeloma cell death in part via ER stress and UPR pathway.^59^

### HIV protease inhibitors

HIV protease inhibitors, such as nelfinavir and atazanavir, have been shown to induce ER stress by increasing the protein load in the ER. In malignant glioma cells, both drugs were shown to cause cell death through stimulation of ER stress response, indicated by increased expression of GRP78 and CHOP and activation of ER stress response-associated caspase-4.^60^ Combination of bortezomib, a proteasome inhibitor, with ritonavir demonstrated enhanced anticancer activity in sarcoma cells, resulting in > 90% apoptosis.^61^ This combination strongly increased the level of ER stress and activated PERK, IRE1, and ATF6, in addition to synergistically inducing CHOP, JNK, caspase-4, and caspase-9, causing irreversible stress and cell death.

### Brefeldin A

Brefeldin A and its prodrug analog breflate are inhibitors of ADP-ribosylation factor and inhibit vesicle trafficking between Golgi and endosomes, leading to accumulation of proteins in ER and subsequent ER stress. Brefeldin A has been reported to trigger apoptosis in several cancer cells, including multiple myeloma, leukemia, colon, and prostate cancers.^6^ In follicular lymphoma cells, brefeldin A-induced apoptosis was associated with profound ER stress that was indicated by GRP78 upregulation and ER dilation, mitochondrial breach, and subsequent caspase cascade activation, including caspase 2 activation.^62^

### GRP78 inhibitors

Versipelostatin specifically inhibits the expression of GRP78, and shows a selective cytotoxicity in glucose-deprived tumor cells *in vitro* and *in vivo*.^63^ Versipelostatin inhibited GRP78 and GRP94 expression and repressed the production of XBP1 and ATF4, leading to massive cell death under glucose deprivation in colon cancer, fibrosarcoma, and stomach cancer cells.

### mTOR inhibitors

Induction of autophagy may be a possible anticancer mechanism of mTOR inhibitors, such as sirolimus, temsirolimus, everolimus, and NV-128, since mTORC1 is a key negative regulator of autophagy. Temsirolimus showed antiproliferative activity in mantle cell lymphoma cells through downregulating p21 and inducing autophagy.^64^ Everolimus increased Beclin1 expression, conversion of LC3-I to LC3-II, and autophagosome formation in acute lymphoblastic lymphoma cells and potentiated the effect of vincristine therapy.^65^,^66^ Everolimus also enhanced sensitivity to radiation therapy through inducing autophagy in breast, lung, and prostate cancer cells.^67^,^68^

### HDAC inhibitors

Histone deacetylase (HDAC) inhibitor, vorinostat, induced both mitochondria-mediated apoptosis and caspase-independent autophagic cell death in HeLa cells.^69^ Other HDAC inhibitors, LAQ824 and LBH589 (panobinostat), also caused autophagic cell death in lymphoma cells when the intrinsic apoptosis pathway was inhibited.^70^ However, HDAC inhibitor-induced autophagy may have a dual role—tumor suppressing and tumor promoting. In glioblastoma cells, vorinostat induced autophagy as a prosurvival mechanism through inhibiting mTOR and upregulating LC3 expression.^71^

### Chloroquine/hydroxychloroquine

Chloroquine and hydroxychloroquine are antimalarial drugs and block lysosomal acidification and degradation of autophagosomes, acting as autophagy inhibitors. These drugs have been shown to have anticancer activities in several cancer cell lines, including breast and colon cancers.^72^,^73^ Since most conventional chemotherapeutic agents induce prosurvival autophagic pathway, a number of phase I/II clinical trials evaluating the combination of hydroxychloroquine and chemotherapeutic agents are ongoing in various cancers, including breast, colon, pancreatic, and lung cancers. A phase III clinical trial in patients with glioblastoma multiforme demonstrated that adding chloroquine to conventional therapy prolonged median survivals from 11 months to 24 months, although the difference was not statistically significant.^74^ In addition to the sensitizing effect to conventional chemotherapy, autophagy inhibition by chloroquine enhanced the anticancer effect of HDAC inhibitors and proteasome inhibitors.^56^,^75^

### Phytochemicals

Phytochemicals, naturally occurring bioactive defense molecules called phytoalexins, have clinical potential in the prevention and treatment of cancer.^76^,^77^ Representative examples include polyphenols such as epigallocatechin-3-gallate (EGCG) from green tea, curcumin from turmeric, and resveratrol from grapes; flavonoids such as quercetin from citrus fruits and genistein from soy; isothiocyanates sulforaphane from broccoli and phenethyl isothiocyanate (PEITC) from turnip and watercress; and organosulfur compounds such as diallyl sulfides from garlic oil.^78^ Despite the body of evidence that phytochemicals might act as ER stress inducers^79^ (e.g., curcumin) or autophagy inducers^80^ (e.g., resveratrol), the mechanisms underlying the beneficial effect of phytochemicals must be further elucidated.

### Others

There are many other classes of UPR/autophagy-targeted drugs that are currently under development and may be worth investigating further for antitumor activity. For example, 2,5-dimethyl-celecoxib (DMC), a structural analog of cyclooxygenase-2 (COX-2) inhibitor, has been found to have all of the antitumor properties of celecoxib, but lack the ability to inhibit COX-2.^6^ Celecoxib is a well-known nonsteroidal anti-inflammatory drug (NSAID) that specifically inhibits COX-2 by blocking the initial step of prostaglandin synthesis. Extensive studies have revealed that the potent antitumor activity of celecoxib might be associated with the induction of ER stress by leakage of Ca^2+^ from the ER into the cytosol. Likewise, DMC was demonstrated to increase intracellular free calcium levels and to induce the UPR pathways by activating GRP78, CHOP/GADD153, and caspase-4. Thapsigargin, an inhibitor of the sarcoplasmic/endoplasmic Ca^2+^ ATPase (SERCA), is another classic ER stress inducer.^81^ Thapsigargin is undergoing preclinical evaluation as a potential targeted agent for prostate cancer.^6^

In addition, tyrosine kinase inhibitors such as imatinib, dasatinib, and sorafenib were shown to induce autophagy in various types of cancer cells.^82^–^84^ Monoclonal antibodies have also been investigated as autophagy inducers. For example, rituximab, a chimeric anti-CD20 monoclonal antibody, might increase intracellular calcium levels and activate calcium/calmodulin-dependent kinase (CaMKK), and then increase autophagy-dependent cell death in lymphoma cells. The anti-EGFR monoclonal antibody panitumumab was shown to increase autophagy via the kinase-independent activity of EGFR in maintaining cancer cell survival. In MCF7 breast cancer cells, the anti-estrogens tamoxifen and toremifen were reported to induce autophagy that was associated with increased resistance to tamoxifen.^85^ Farnesyltransferase inhibitors such as lonafarnib and poly(ADP-ribose) polymerase inhibitors like ABT-888 are other classes of drugs that have been associated with inducing autophagy in osteosarcoma cell line and non-small cell lung cancer cells, respectively.^86^,^87^ Furthermore, other compounds being investigated as potential autophagy inducers include vitamin D analog EB1089,^88^ Bcl-2 inhibitor GX15–070,^89^ and 2-deoxyglucose,^90^ and potential autophagy inhibitors include quinacrine^91^ and 3-methyladenine.^85^

## Conclusion and perspectives

Many of the signaling pathways related to cellular stress responses such as the UPR and autophagy appear to be associated with cell fate decisions in tumors growing under stressful conditions. Nevertheless, the complex mechanisms of action of UPR/autophagy-targeted agents have not yet been fully elucidated. We cannot predict whether these targeted agents actually induce or inhibit the stress response pathways. Despite the uncertainty of how the modulation of the UPR and autophagy pathways affect cell fate, there are a number of compounds currently in preclinical and clinical development that target processes that have a direct impact on the UPR and autophagy in cancer.

However, there are two major challenges for targeting cellular stress responses in cancer. One is finding a therapeutic window where it is possible to selectively kill cancer cells without harming normal cells. The other is developing good biomarkers to measure and evaluate the dynamic stress responses for the selection and follow-up of the patients. Further clinical and experimental studies are essential to successfully meet these challenges and to understand the mechanisms regulating the cellular stress responses through the intra- and extracellular signaling networks.
